# Neurological manifestations of Mpox virus during the recent global outbreak: a systematic review

**DOI:** 10.1186/s12879-025-11631-w

**Published:** 2025-09-29

**Authors:** Govinda Bhandari, Anush Acharya, Anupam Katuwal Chettri, Sujan Sharma

**Affiliations:** 1 Institute of Medicine, Maharajgunj Medical Campus, Kathmandu, Nepal; 2 Kushmisera Primary Health Center, Baglung, Nepal

**Keywords:** Monkeypox, Mpox virus, Neurological manifestations, Brain, Systematic review

## Abstract

**Introduction:**

Mpox is a zoonotic infection caused by the Mpox virus (MPXV), an Orthopoxvirus known to cause fever, lymphadenopathy, mucocutaneous lesions, and some systemic complications. With the recent global outbreak, an increasing number of neurological manifestations have been reported. This systematic review aims to assess and summarize the neurological manifestations associated with Mpox virus infection, highlighting its prevalence, frequency, and clinical impact.

**Methods:**

Studies that included data from recent outbreaks, published between May 2022 to January 2025 that reported neurological manifestations in patients infected by Mpox were systematically reviewed using the PRISMA (Preferred Reporting Items for Systematic Review and Meta-Analysis) statement.

**Results:**

Our systematic review included data from 20 studies encompassing data from 2,245 individuals across multiple regions. Neurological manifestations ranged from common symptoms such as fatigue (32.6%), myalgia (29.7%), and headache (29.2%) to severe complications like encephalitis (0.23%), encephalomyelitis (0.23%), transverse myelitis (0.09%), Guillain-Barré Syndrome (0.045%), and Parsonage-Turner Syndrome (0.045%). Autonomic nervous system involvement, including bowel and bladder incontinence, was reported in 0.26% of cases. Severe cases required immunomodulatory therapies such as corticosteroids, intravenous immunoglobulin (IVIG), and antiviral treatments. Recovery varied, from a few weeks, with some patients experiencing prolonged symptoms lasting up to 32 weeks, and a few reporting persistent deficits.

**Conclusion:**

This systematic review highlights the wide spectrum of neurological complications associated with Mpox infection, ranging from mild symptoms to severe, debilitating conditions. While most patients experienced resolution of symptoms with appropriate management, a subset developed long-term deficits, underscoring the need for vigilant monitoring and early intervention. Given the increasing global incidence of Mpox, healthcare professionals must be aware of its potential neurological impact to ensure timely diagnosis and effective treatment.

## Introduction

Mpox virus (MPXV), formerly known as Monkeypox virus is a zoonotic infection caused by infection with monkeypox virus (MPXV), an Orthopoxvirus(OPXV) [1]. The *Orthopoxvirus* genus contains other viruses, including the variola virus and vaccinia virus, that cause diseases such as smallpox, cowpox, horsepox, and camelpox [2]. Squirrels, dormice, Gambian giant rat, hedgehog, jerboa, opossum, and woodchucks are the reservoirs of the virus [3], and infection of humans and monkeys appears to be incidental [4]. The viruses are transmitted through direct inoculation by bites and scratches or by direct contact with bodily fluids, particularly when hunting or playing with the infected animals [4]. Human-to-human transmission is less common and occurs primarily via respiratory droplets or by direct contact with lesions’ secretions [5]. 

The incubation period of MPXV ranges between 5 and 21 days, and clinical features are malaise, fever, rigors, headache, generalized lymphadenopathy, and centrifugal pattern of skin rash mainly on the face, palms, and soles, starting one to three days after fever onset [6]. The illness is typically self-limiting, but in some cases, including younger age, a person having underlying immune deficiencies like HIV and other chronic illnesses, the disease may complicate and can cause bronchopneumonia, sepsis, encephalitis, and corneal involvement, causing loss of vision [7]. Neurological manifestation ranges from nonspecific symptoms such as headache, myalgia, fatigue, and photophobia to severe complications such as seizure and encephalitis [8]. The mechanisms of neuroinvasion in humans are unclear, but in animal studies done by Kulesh et al. [9], mpox has demonstrated evidence of neuroinvasion, capable of crossing the blood-brain barrier either through the olfactory epithelium or via infected monocytes and macrophages. Although routes of transmission to the CNS is not totally known, in animal studies, two possible mechanisms have been speculated: through the olfactory pathway and the macrophage monocyte pathway [10]. 

Although endemic in West and Central Africa, many cases of Mpox are being seen internationally. The first case outside Africa was reported from the USA in 2003. Between 2018 and 2021, a number of cases have been reported from some countries (1 in Israel,1 in Singapore,& in the UK): 5 were in returning travelers from Nigeria [7]. Since 2022, the exponential increase in cases of Mpox and in people with no history of travel to endemic areas has been seen. In the period between 1 st January and 22nd July 2022, 16,016 laboratory-confirmed cases and 5 deaths have been reported to WHO from 75 countries in all six WHO regions, with the maximum cases from Spain(3125) followed by the United States of America(2316) [7]. 

As per WHO data, as of 29 April 2025 reported a total of 137,892 confirmed cases of monkeypox worldwide from 1 January 2022 to 31 March 2025, with 317 deaths documented across 132 countries [11]. This review highlights the increasing neurological manifestations of Mpox in recent outbreaks and the need for further research to determine the prevalence, frequency, and long-term outcome of such complications.

## Methodology

Our systematic review was registered in Prospero (ID CRD42025642739). The systematic review was performed in accordance with the PRISMA (PREFERRED Reporting Items for Systematic Reviews and Meta-Analysis) statement in conjunction with the PRISMA checklist and flow diagram for manuscript format development [12]. 

### Literature Search

A comprehensive literature search was conducted to identify studies reporting neurological manifestations of the Mpox virus (MPXV) during recent global outbreaks. The search strategy was independently developed by the authors, noting that no librarian was involved and that the search was not peer-reviewed using PRESS. Potentially relevant studies were identified through a systematic search of PubMed and Google Scholar databases. The search strategy combined Medical Subject Headings (MeSH) terms and free-text keywords using Boolean operators, truncation, and filters. The full PubMed search string was as follows: (“Mpox virus“[MeSH Terms] OR “Mpox virus” OR “Monkeypox virus”) AND (neurological OR brain OR encephalitis OR seizures OR neuropathy OR “neurological manifestations”) AND (manifestations OR symptoms OR complications) AND (outbreak OR pandemic OR global).

The search was applied to the title, abstract, and keywords fields. Language restrictions was applied to English only and Limited to studies published between May 2022 and January.The records found through database searching were combined and the duplicates were removed using Covidence. The study initially screened all unique published articles based on title and abstract, and Further filtered out by reviewing Full articles considering inclusion and exclusion criteria.This approach ensures reproducibility and transparency of the literature search in line with PRISMA 2020 guidelines.

### Eligibility Criteria

Studies were included if they involved individuals diagnosed with Mpox virus infection, whether confirmed or suspected, with a specific focus on the presence of neurological manifestations. Eligible study designs encompassed case reports, case series, observational studies, clinical trials, and randomized controlled trials. Only literature published in English between May 2022 and January 2025 was considered. Conversely, studies were excluded if they did not address neurological outcomes, if they were animal studies, editorials, or non-peer-reviewed sources, or if they were published in languages other than English or prior to May 2022.

### Data Extraction

The data extraction was done by two researchers (GB and AK) independently from included studies using a standardized and pretested format prepared in Microsoft Excel. Both the initial title/abstract screening and the full-text screening were conducted independently by two reviewers and discrepancies were resolved by discussion with a third reviewer. A detailed review of each article was done to gather information about the author, journal name, publication year, country, WHO region, type of study, sample size, age, percentage of males and females, HIV status, neurological manifestations, onset, duration, resolution, and treatment. The information was recorded in Google Sheets. Two researchers, GB and AK performed data extractions separately to negotiate any possible errors. Disagreement on data extraction between researchers was resolved through discussion and consensus. The extracted data were checked at least twice for their accuracy.

### Outcome measures

Our outcome was to elucidate the neurological manifestations of Mpox reported in the medical literature. The results were divided into four categories: common neurological symptoms, central nervous system manifestations, peripheral nervous system, and autonomic nervous system involvement.

## Results

### Study Characteristics

**Fig. 1:**
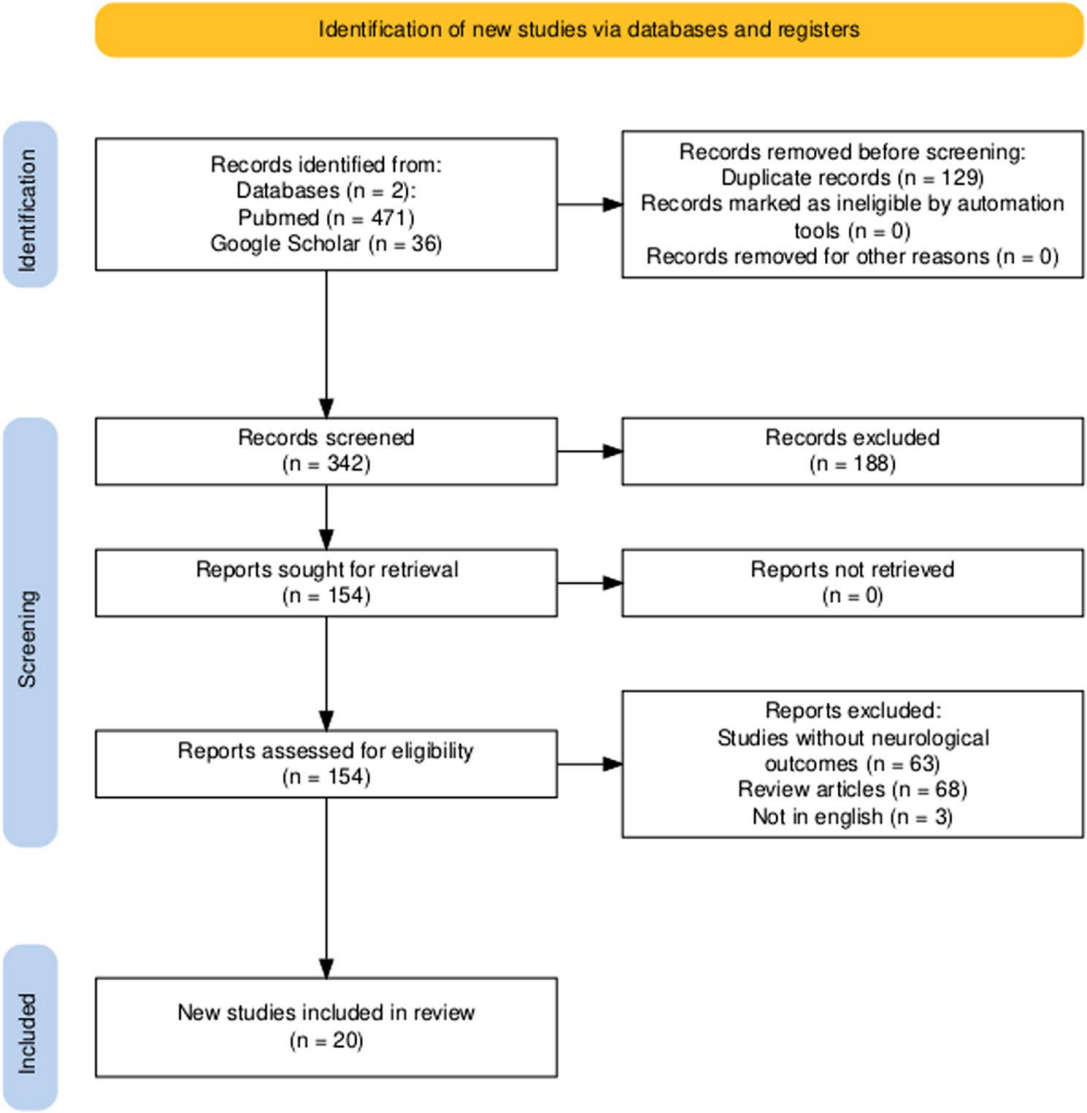
PRISMA flow diagram depicting the flow of information through the different phases of a systematic review

In total, our literature search yielded 507 articles. After excluding duplicates and those not meeting the inclusion criteria, only 20 papers focusing on neurological symptoms associated with Mpox were included in our systematic review. (Fig. 1).

These studies span across different countries five from the USA [13–17], two from the UK [18, 19], and one from China [20], Saudi Arabia [21], Sweden [22], Peru [23], UAE [24], Switzerland [25], Columbia [26], Belgium [27], Brazil [28], Italy [29], and Korea [30]. Eight studies were from the Region of the Americas and six from the European Region, two from the Eastern Mediterranean region, and two were from the Western Pacific region, covering a mix of case reports [9], case series [4], cohort studies [5], and one cross-sectional study. The sample sizes vary significantly, ranging from single case reports to large cohort studies, with the highest sample size being 928 in a study from Brazil [28]. It also reported a case series from 16 different countries and one cohort study from 29 different countries [31, 32]. The characteristics of each study are summarized in Table 1.

**Table 1:** Study information

Author	Journal	Publication Year	Country	WHO Region	Type of Study	Sample Size
Fu (13)	Journal of Medical Virology	2023	China	Western Pacific Region	Cross-sectional	115
Moore (14)	Journal of Immunology	2023	USA	Region of the Americas	Case report	1
Hammad (15)	Cureus	2025	Saudi Arabia	Eastern Mediterranean Region	Case report	1
Karin (16)	BMC Infectious Disease	2024	Sweden	European Region	Case report	1
Rodríguez (17)	Emergency Radiology	2023	Peru	Region of the Americas	Case report	1
Pastula (18)	MMWR	2022	USA	Region of the Americas	Case series	2
AbdulHamid (19)	Case Report in Infectious Disease	2023	UAE	Eastern Mediterranean Region	Case report	1
Gerber (20)	BMJ Case Reports	2024	Switzerland	European Region	Case report	1
Sharma (21)	Cureus	2023	USA	Region of the Americas	Case report	1
Marín-Medina (22)	Journal of Medical Virology	2023	Colombia	Region of the Americas	Case report	1
Cole (23)	Lancet Infectious Disease	2023	UK	European Region	Case report	1
Money (24)	Annals of Neurology	2023	USA	Region of the Americas	Case series	3
Charniga (25)	medRxiv (preprint)	2022	USA	Region of the Americas	Cohort	21
Thornhill (26)	New England Journal	2022	16 countries	-	Case series	538
Hens (27)	New Microbes New Infect	2023	Belgium	European Region	Cohort	116
Patel (28)	BMJ	2022	UK	European Region	Cohort	197
Ribeiro (29)	Epidemiol Serv Saude	2024	Brazil	Region of the Americas	Cohort	928
Gaspari (30)	Journal of Clinical Microbiology	2023	Italy	European Region	Case series	30
Lim (31)	Journal of Korean Medical Science	2024	Korea	Western Pacific Region	Cohort	60
Angelo (32)	Lancet Infectious Disease	2023	29 countries	-	Cohort	226

A total of 2,245 individuals diagnosed with Mpox virus infection from 20 different studies were included in our systematic review.

The studies were published in various medical journals between 2022 and 2025, indicating that research on this topic is recent and evolving. Notably, high-impact journals such as New England Journal of Medicine [31], Lancet Infectious Disease [18, 32], and Annals of Neurology [16] have featured relevant studies, highlighting the significance of neurological manifestations in Mpox cases.

Geographically, the studies originate from multiple regions, including North America, South America, Europe, Asia, and Africa, ensuring a diverse representation of Mpox cases and their neurological presentations. Some studies analyze multi-country data, with one involving cases from 29 different countries.

### Demographic Characteristics

The mean age of the reported cases ranges between 27 and 38 years, with a few studies reporting broader age distributions (e.g., 0–93 years in a Brazilian cohort). The majority of cases involve male patients, with some studies exclusively reporting on males (100%). Female representation is Limited, with a few studies reporting up to 6.3% female patients. Out of 2245 individuals, only 72 were female. (Table 2)

**Table 2:** Demographic characteristics and disease information

Author	Age	Male %	Female %	Comorbidities	Neurological Symptoms	Duration of symptoms	Recovery
Fu (13)	Range 27-36.5 Mean 31	100	0	HIV Positive 65	Myalgia 28	N/A	N/A
Moore (14)	36	100	0	HIV Positive 1	Transverse myelitis 1,Fatigue 1, Limb weakness 1,Limb numbness 1	20 weeks	Not Recovered
Hammad (15)	31	100	0	0	Encephalomyelitis 1,Limb weakness 1, Limb numbness 1, Bladder Incontinence 1, Facial paralysis 1,Confusion 1	4 weeks	Complete Recovery
Karin (16)	37	100	0	Active Syphillis 1	Encephalitis 1,Confusion 1,Fatigue 1,Headache 1	4 weeks	Complete Recovery
Rodríguez (17)	30	100	0	0	Encephalomyelitis 1,Limb weakness 1,Limb numbness 1, Confusion 1	N/A	N/A
Pastula (18)	30	100	0	0	Encephalomyelitis 2,Limb weakness 2,Limb numbness 1, Bladder Incontinence 2	4–5 weeks	Assistive Walking
AbdulHamid (19)	53	100	0	0	Guillain-Barre Syndrome 1,Limb weakness 1, Bladder Incontinence 1)	N/A	N/A
Gerber (20)	30	100	0	Epilepsy 1, Hep C 1	Parsonage-Turner syndrome 1	32 weeks	Complete Recovery
Sharma (21)	44	100	0	0	Encephalitis 1,Confusion 1	N/A	N/A
Marín-Medina (22)	30			N/A	Encephalomyelitis 1,Slurred speech 1	3 weeks	Complete Recovery
Cole (23)	35	0	100	0	Encephalitis 1,Transverse myelitis 1	12 weeks	Complete Recovery
Money (24)	30	100	0	0	Limb weakness 3,Limb numbness 1, Bladder/Bowel retention 2,Headache 1, Encephalitis 2,Seizure 1,Confusion 2	8 weeks 1,4 weeks 2	Complete Recovery 2,Impaired Gait 1
Charniga (25)	Range 28–61 Median 37	100	0	N/A	Headache 21,Myalgia 21	N/A	N/A
Thornhill (26)	Mean 38	98	2	HIV Positive 216	Headache 145,Myalgia 167,Fatigue 220	N/A	N/A
Hens (27)	N/A	0	100	HIV Positive 53	Headache 35,Myalgia 49,Fatigue 62	N/A	N/A
Patel (28)	Mean 38	93.7	6.3	N/A	Headache 49,Myalgia 62,Fatigue 46	N/A	N/A
RibeiroCLP (29)	Range 0–93,Mean 34	93.7	6.3	HIV Positive 389, Active syphilis 790	Headache 359,Myalgia 268,Fatigue 286	N/A	N/A
Gaspari (30)	Mean 37.5	100	0	HIV Positive 12	Myalgia 17,Fatigue 17	N/A	N/A
Lim (31)	Range 21–58, Mean 32	97	3	HIV positive 25, DM, 2, Hypertension, 2 HBV infection, 1	Headache 11,Myalgia 23,Fatigue 7	N/A	N/A
Angelo (32)	Range 18–68,Mean 37	100	0	HIV Positive 92	Headache 35,Myalgia 32,Fatigue 92	N/A	N/A

Comorbidities were frequently reported, with HIV being the most common, especially in large cohort studies, up to 41.9% (389 cases) in a Brazilian study [28]. Among the studies that reported HIV status, a total of 853 were HIV-positive patients were identified. Other notable comorbidities include active syphilis, hypertension, diabetes mellitus, hepatitis B, hepatitis C, and epilepsy [22, 25, 28, 30]. 

### Neurological Manifestation

**Fig. 2:**
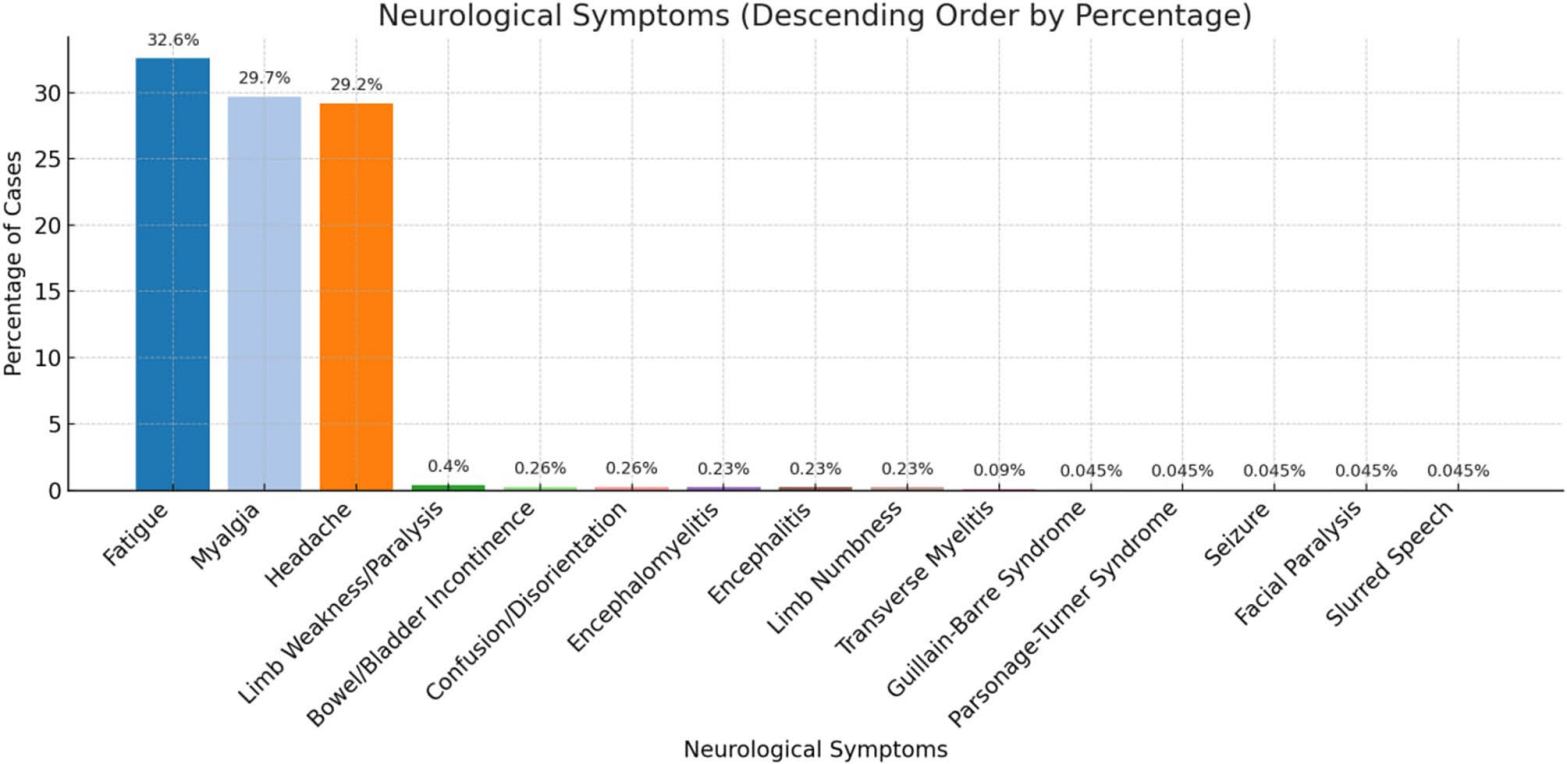
Different Neurological Manifestation of Mpox

Various neurological manifestations were reported, ranging from common symptoms like headache and myalgia to rare but severe conditions such as encephalitis, transverse myelitis, and Guillain-Barré Syndrome which are summarized in Fig. 2. Confirmed neurological manifestations included seizures, encephalitis, neuropathies, and Guillain–Barré syndrome, which directly indicate involvement of the central or peripheral nervous system. In contrast, symptoms such as headache, fatigue, myalgia, and photophobia, while frequently reported, are nonspecific manifestations common to many viral infections and do not necessarily reflect direct neurological involvement.

### Common Neurological Symptoms

#### Fatigue

Fatigue was reported in 732 individuals (32.6%), making it the most common neurological manifestation. This symptom likely reflects a combination of viral illness, immune activation, and possible central nervous system involvement.

#### Myalgia

Myalgia was observed in 667 cases (29.7%). Muscle pain was often diffuse and accompanied by generalized fatigue, suggesting a systemic inflammatory response to viral infection.

#### Headache

Headache was one of the most frequently reported neurological symptoms, affecting 657 individuals (29.2%). It often appeared in the early stages of infection and was commonly associated with fever and fatigue. The underlying mechanism remains unclear but could be related to viral-induced inflammation or systemic immune responses.

### Central Nervous System Involvement

#### Encephalitis

Encephalitis, a severe inflammatory condition affecting the brain, was documented in 5 cases (0.23%) [15, 16, 18, 22]. Patients presented with altered mental status, confusion, and in some cases, seizures. The pathophysiology of Mpox virus-related encephalitis remains unclear, but direct viral invasion or immune-mediated mechanisms are potential contributors.

#### Encephalomyelitis

Encephalomyelitis was observed in 5 individuals (0.23%) [14, 21, 23, 26]. This condition, characterized by inflammation of both the brain and spinal cord, can lead to motor and sensory deficits. Clinical presentations varied from confusion and weakness to autonomic dysfunction, suggesting multifocal involvement.

#### Transverse myelitis

Transverse myelitis, a rare inflammatory disorder of the spinal cord, was identified in 2 cases (0.09%) [13, 18]. Affected individuals experienced limb weakness, numbness, and bowel or bladder dysfunction. The presence of myelitis in Mpox infection suggests a potential post-infectious autoimmune response.

#### Confusion and disorientation

Confusion or disorientation was observed in 6 individuals (0.26%) [15, 16, 21–23]. These symptoms were likely linked to encephalitis, encephalomyelitis, or systemic inflammatory responses affecting brain function.

#### Seizures

Seizures occurred in 1 individual (0.045%) [16]. This rare manifestation could be attributed to direct viral neuroinvasion, systemic inflammation, or metabolic disturbances related to Mpox infection.

### Peripheral Nervous System Involvement

#### Guillain-Barré syndrome (GBS)

GBS was diagnosed in 1 case (0.045%),based on acute bilateral limb weakness with areflexia, CSF showing albuminocytologic dissociation (protein 106 mg/dl with normal glucose and no pleocytosis), supportive MRI findings of cauda equina root enhancement, and exclusion of other causes, in the setting of recent monkeypox infection [24]. This immune-mediated disorder leads to ascending paralysis, areflexia, and autonomic dysfunction. While viral infections are known triggers of GBS, their association with the Mpox virus remains unclear and warrants further study.

#### Parsonage-Turner syndrome

Parsonage-Turner Syndrome, a rare inflammatory neuropathy affecting the brachial plexus, was reported in 1 individual (0.045%) [25]. The patient experienced acute-onset shoulder pain followed by progressive weakness, suggesting an immune-mediated mechanism.

#### Limb weakness

Limb weakness or paralysis was observed in 9 individuals (0.4%) [13, 21]^23^– [16, 24]. The underlying causes varied, including direct viral effects, immune-mediated inflammation, and post-infectious complications like myelitis or GBS.

#### Limb numbness

Limb numbness was reported in 5 cases (0.23%) [13, 14, 16, 21, 23]. Sensory deficits were often associated with other neurological symptoms, including weakness and autonomic dysfunction.

#### Facial paralysis

Facial paralysis was documented in 1 case (0.045%) [21]. While the exact mechanism remains unclear, viral infections have been associated with cranial nerve involvement, leading to conditions such as Bell’s palsy.

#### Slurred speech

Slurred speech was noted in 1 individual (0.045%) [26], possibly resulting from central or peripheral nervous system involvement. This symptom may be associated with encephalitis, myelitis, or cranial nerve dysfunction.

### Autonomic Nervous System Involvement

#### Bowel and bladder incontinence

Bowel and bladder incontinence was reported in 6 cases (0.26%) [14, 16, 21, 24], indicating possible involvement of the spinal cord or autonomic nervous system. These symptoms were often associated with transverse myelitis and other spinal cord-related complications.

### Treatment Approaches

The management of neurological manifestations associated with Mpox virus infection varied based on severity. Common symptoms such as headache, myalgia, and fatigue were managed with symptomatic treatment, including analgesics, antipyretics, and supportive care. More severe neurological complications like encephalitis, encephalomyelitis, and transverse myelitis required proper monitoring and targeted therapies. High-dose intravenous (IV) methylprednisolone (1 g/day) was commonly administered, often followed by intravenous immunoglobulin (IVIG) at a dose of 0.2 g/kg or 0.4 g/kg [13, 24]. Some cases received plasma exchange as an adjunct therapy [14]. Antiviral agents such as brincidofovir and IV tecovirimat were administered [14]. Patients with severe or prolonged weakness also underwent physical therapy to aid in functional recovery [25]. 

### Onset and Resolution of Symptoms

The common neurological symptoms such as headache, myalgia, and fatigue are often reported early in the infection. All other neurological symptoms are acute on onset and most of them appear at the active phase or late phase of Mpox infection. In Pastula et al. [14] the symptoms of encephalomyelitis appeared after 9 days of other symptoms and after 2 weeks the symptoms of PTS appeared [25]. Among the studies that reported symptom duration, the values ranged from 3 weeks to 32 weeks. Several studies indicated that patients recovered within 4 to 5 weeks [14, 16, 21, 22, 26], while others reported more prolonged durations, such as 12 weeks [18], 20 weeks [13], and even 32 weeks [25]. (Overall, these findings highlight the heterogeneity in the course of neurological symptoms among Mpox patients.

Outcomes varied among affected individuals. While most of the patients achieved complete recovery [18, 21, 22, 25, 26], others experienced persistent neurological deficits. However, some individuals required assistive walking devices due to residual weakness [14]. A few cases did not recover [13], while some with persistent impairments such as impaired gait [14]. These findings highlight the variability in disease progression and underscore the need for long-term follow-up in patients with Mpox virus-associated neurological complications.

## Discussion

Globally, Mpox has become a threat as there are more reported cases; in some countries, many more than before, and in some for the first time as new cases. Since 1970, 10 African countries have reported cases of Mpox virus (MPXV), and the maximum number of cases have been reported from the Democratic Republic of Congo, followed by Nigeria [7]. In DRC, the number of Mpox cases has increased more than tenfold over the past five decades, with a significant increase in recent years [33]. People are seriously thinking about Mpox, though it is less severe than the previous one, it is because of the terrified mental status of people after the COVID-19 pandemic. The 2022 monkeypox outbreak has affected 110 countries worldwide, outside of classic endemic areas (i.e., West Africa and Central Africa). The outbreak was declared a public health emergency of international concern by the WHO on July 23, 2022 [18]. Although less severe than some historical outbreaks, the widespread transmission and public concern highlight the need for awareness and preparedness. This systematic review provides an overview of the neurological symptoms of the recent global outbreak of Mpox infection. We have only included the articles that were published after the recent outbreak of Mpox from May 2022.

The 2022 mpox outbreak was characterized by dominant clinical features of skin lesions, fever, and lymphadenopathy, with anogenital lesions and inguinal lymphadenopathy being the most common, along with concerns over fatigue, headache, and myalgia [34]. Within this context, neurological manifestations, though less frequent, represent an important aspect of disease morbidity. In our analysis, the findings suggest that the vast majority of cases are in males, and a substantial number of these patients are also HIV-positive. This dual burden highlights the importance of considering co-infection dynamics and potential implications for disease severity and management strategies in this population. There is a wide range of symptoms, but major neurological symptoms are relatively uncommon. Immune status appears to influence the spectrum and severity of mpox disease. Symptoms vary according to the patient’s status as immunocompetent or immunocompromised. Symptoms like mucocutaneous rash, fever, lymphadenopathy, headache, generalized malaise, pruritus, bronchopneumonia, cough, arthralgia, oropharyngitis, proctitis, and depression/anxiety are more common in immunocompetent patients. However, fever, headache, myalgia, lymphadenopathy, pericarditis, myocarditis, ophthalmological problems, adrenal insufficiency, and neurological symptoms are more common among immunocompromised patients [18, 31]^35^– [38]. This observation suggests that immunocompromised individuals, particularly those with HIV infection, may be at higher risk of severe neurological complications due to impaired immune responses, higher viral loads, or increased susceptibility to secondary infections. However, the current literature is limited, and most evidence derives from small case reports or series, which prevents definitive conclusions. Understanding the influence of immune status on neurological outcomes is clinically important, as it may guide monitoring and management strategies. Future prospective studies are needed to systematically assess the impact of immunosuppression on the severity, frequency, and prognosis of neurological manifestations associated with Mpox virus infection.

There are very few articles that mention the neurological symptoms and complications in patients infected with the Mpox virus. Common neurological problems in patients include confusion or altered mental status, encephalitis, limb weakness, and numbness. Some researchers also mention the general prodromal symptoms of viral illness, like myalgia, headache, and fatigue also as neurological symptoms, for effective reporting and comparison [39]. Along with these common systemic manifestations, potentially life-threatening conditions of the central nervous system like encephalitis, encephalomyelitis, and transverse myelitis, are also reported in the patients. Only one case had experienced a seizure, whose pooled prevalence was 2.7% in the previous outbreak [16]. Similarly, symptoms of photophobia and coma were not reported in this outbreak, which were seen in earlier outbreaks [39]. Although less frequently assessed, the cases of Guillain-Barré syndrome, Parsonage-Turner syndrome, limb weakness, limb numbness, facial paralysis, and slurred speech are also reported as the virus or its antigen or antibodies against it involve the peripheral nervous system, which has not been reported before this outbreak [21, 24–26]. Some cases of bowel and bladder incontinence are reported, indicating the involvement of the autonomic nervous system [14, 16, 21, 24]. This symptom is likely associated with transverse myelitis or spinal cord involvement in patients infected with mpox. Outcomes of mpox-associated neurological complications vary, with most patients recovering within 1–2 months, while some experience persistent deficits like impaired gait or require assistive devices. A case of Parsonage-Turner syndrome took 8 months to recover, highlighting the variability in disease progression and the need for long-term follow-up.

The underlying mechanisms for these neurological manifestations are not yet fully understood. Potential explanations include direct central nervous system (CNS) infection by the virus, immune-mediated responses, and psychological reactions to the illness [33]. Evidence from animal studies suggests that MPXV and related orthopoxviruses may access the central nervous system through multiple routes, including trans-synaptic spread via the olfactory pathway and hematogenous dissemination through infected monocytes and macrophages crossing the blood–brain barrier [9, 10]. These mechanisms highlight the biological plausibility of direct neurological involvement in human Mpox infections, although definitive evidence in clinical settings remains limited. This has important implications for clinical practice, as it may underlie the occurrence of severe complications such as encephalitis, seizures, or acute neuropathies in affected patients,

Relevant history from patients, course of progression of symptoms make us think about mpox infection. Real-time PCR for DNA detection of mpox from cutaneous lesion swab, serum, and oropharyngeal swab is the gold standard for diagnosis of mpox infection. There may not be detection of DNA of mpox in CSF of patients complicated with CNS involvement but antibodies to Mpox may be reported and elevated protein levels can be seen in CSF [34]. Neuroimaging, particularly MRI, helps identify brain abnormalities consistent with encephalitis or other neurological conditions involving CNS and PNS [34]. Serology investigation for HIV, Hepatitis B, and Hepatitis C is necessary as mpox infection and prevalence of complications are reported more in HIV positive cases and is important for understanding comorbidity and immune status [26, 30].

Managing neurological complications in mpox involves a combination of antiviral treatments, supportive care, and, in certain cases, immunomodulatory therapies. Mpox virus (MPXV) infections do not currently have a particular treatment; however, tecovirimat, brincidofovir, cidofovir, and vaccinia intravenous immunoglobulin are pharmacological therapies that have been approved for compassionate use and can be used to treat severe cases [23, 24]. Supportive care is essential and may include measures such as intravenous fluids, pain management, and monitoring for potential complications. Steroid and intravenous immunoglobulin, along with antiviral therapy, have good outcomes among patients with complications as it decreases the flare of inflammation in the brain and spinal cord [13, 14, 24]. Plasmapheresis might be considered, especially if there’s evidence of immune-mediated neurological damage [25, 40]. Most of the complication cases are managed almost similarly in our analysis with antiviral therapy along with steroids and intravenous immunoglobulin.

While headache, myalgia, and fatigue were the most common neurological symptoms, our review highlights the occurrence of rare but serious complications, including encephalitis, transverse myelitis, and Guillain-Barré Syndrome. Although most of the conditions are self-resolving and will recover, it may be fatal if not diagnosed and treated on time [41], few may not recover and have reported enduring impairments and residual symptoms.A broad vision on clinical symptoms and analysis on it will lead to proper diagnosis before any complications occur, thus there will be no more seriousness about the disease and no more transmission among people if preventative measures are applied.

This systematic review has several limitations. The number of published cases reporting neurological manifestations of the Mpox virus (MPXV) is relatively small and largely based on single-patient case reports or small case series, leading to heterogeneity in study design, sample size, patient demographics, and geographic settings. Combining data from such diverse studies may overstate comparability and limit the reliability of prevalence estimates. Some symptoms reported in the included studies, such as headache, fatigue, and myalgia, are common systemic manifestations of viral infections and may not reflect direct neurological involvement. By distinguishing these from confirmed neurological symptoms, we aimed to provide a more accurate representation of the neurological burden of Mpox virus infection. In addition, the literature search was restricted to two databases (PubMed and Google Scholar), Limited to studies published in English between May 2022 and January 2025, and the search strategy was not peer-reviewed, which may have resulted in missing relevant studies. Finally, due to heterogeneity and the descriptive nature of the available data, a meta-analysis was not feasible, and the findings and results should be interpreted as a qualitative synthesis.

## Conclusion

This systematic review provides a comprehensive analysis of the neurological manifestations of Mpox virus infection during the recent global outbreak, emphasizing both common symptoms such as fatigue, myalgia, and headache and rarer but severe complications like encephalitis, transverse myelitis, seizure, facial palsy and Guillain-Barré Syndrome. Though rare, it underscores the virus’s potential neurotrophic effects with a broader spectrum of neurological complications than previously documented. While most patients recovered within 1–2 months, some experienced persistent neurological deficits, requiring long-term rehabilitation and follow-up. Given the increasing incidence of Mpox beyond endemic regions, early recognition of neurological symptoms, improved diagnostic strategies, and targeted treatment approaches—including antiviral therapy, corticosteroids, IV immunoglobulin, and supportive care—are crucial for better clinical outcomes. Further research is needed to determine the long-term neurological impact of Mpox and to develop more effective therapeutic interventions.

## Data Availability

All data generated or analyzed during this study are included in this published.

## Abbreviations

MPXV: Mpox virus
CNS: Central Nervous System
CSF: Cerebrospinal Fluid
DM: Diabetes Mellitus
GBS: Guillain-Barré Syndrome
HBV: Hepatitis B Virus
HCV: Hepatitis C Virus
HIV: Human Immunodeficiency Virus
IV: Intravenous
IVIG: Intravenous Immunoglobulin
MRI: Magnetic Resonance Imaging
N/A: Not Applicable
OPXV: Orthopoxvirus
PCR: Polymerase Chain Reaction
PNS: Peripheral Nervous System
PRISMA: Preferred Reporting Items for Systematic Reviews and Meta-Analysis
PTS: Parsonage-Turner Syndrome
USA: United States of America
WHO: World Health Organization
